# Passive and active ventricular elastances of the left ventricle

**DOI:** 10.1186/1475-925X-4-10

**Published:** 2005-02-11

**Authors:** Liang Zhong, Dhanjoo N Ghista, Eddie YK Ng, Soo T Lim

**Affiliations:** 1 College of Engineering, School of Mechanical and Production Engineering, Nanyang Technological University, 50 Nanyang Avenue, Singapore 639798; 2 Bioengineering Division, College of Engineering, Nanyang Technological University, Singapore, 639798; 3 Department of Cardiology, National Heart Centre, SingHealth, Mistri Wing, 3^rd ^Hospical Ave., Singapore, 168572

## Abstract

**Background:**

Description of the heart as a pump has been dominated by models based on elastance and compliance. Here, we are presenting a somewhat new concept of time-varying passive and active elastance. The mathematical basis of time-varying elastance of the ventricle is presented. We have defined elastance in terms of the relationship between ventricular pressure and volume, as: *dP *= *EdV *+ *VdE*, where E includes passive (E_p_) and active (E_a_) elastance. By incorporating this concept in left ventricular (LV) models to simulate filling and systolic phases, we have obtained the time-varying expression for E_a _and the LV-volume dependent expression for E_p_.

**Methods and Results:**

Using the patient's catheterization-ventriculogram data, the values of passive and active elastance are computed. E_a _is expressed as: ; E_p_is represented as: . E_a _is deemed to represent a measure of LV contractility. Hence, Peak dP/dt and ejection fraction (EF) are computed from the monitored data and used as the traditional measures of LV contractility. When our computed peak active elastance (E_a,max_) is compared against these traditional indices by linear regression, a high degree of correlation is obtained. As regards E_p_, it constitutes a volume-dependent stiffness property of the LV, and is deemed to represent resistance-to-filling.

**Conclusions:**

Passive and active ventricular elastance formulae can be evaluated from a single-beat P-V data by means of a simple-to-apply LV model. The active elastance (E_a_) can be used to characterize the ventricle's contractile state, while passive elastance (E_p_) can represent a measure of resistance-to-filling.

## Background

The heart may be conceived as a pump that receives blood from a low-pressure system and raises it to a high-pressure system. Although the mechanism responsible for generation of wall stress (and hence left ventricular (LV) pressure) is contraction of the myocardial fibers, an analytical formulation linking the myocardial and LV dynamics is still lacking. In the absence of this formulation, a popular way of linking LV pressure and volume dynamics is by means of LV compliance (or elastance) [1,2]. Although this yields the cyclic values of elastance and compliance, this concept does not provide an intrinsic measure of elastance and compliance for the contractile state of the LV.

The concept of compliance or elastance was first employed for blood vessels [3], by relating incremental cross-section area (or volume) and transmural pressure. Warner appears to be have been the first to adopt a compliance description for a dynamic heart [4]. In Warner's description, a mean value of compliance is adopted for diastolic phase and another mean value for systole, with abrupt transitions between the two states.

Defares [5] avoided the stepwise transition between diastolic and systolic compliance, by making elastance a continuously varying function of time. Later, the concept of a continuously varying compliance or elastance was adopted by a number of investigators with diverse variations [2,6,7]. Nevertheless, they all share the concept of a simple and extrinsic compliance term as an adequate description of ventricular mechanics during the cardiac cycle, based on monitored values of LV pressure and volume.

Classically, ventricular compliance is defined, at any point in time, as the change in ventricular volume concomitant with the change in ventricular pressure, such that

*C *= *dV */ *dP*, *E *= *dP */ *dV *    (1)

If C is assumed constant, this equation becomes a linear relation, whose integration gives

*V *= *CP *+ *V*_*c *_    (2)

where V_c _is an integration constant. If the compliance varies with time, then all terms in equation (2) may vary with time, as:

*V *(*t*) - *V*_*c *_(*t*) = *C*(*t*)*P*(*t*)     (3)

**Figure 1:**
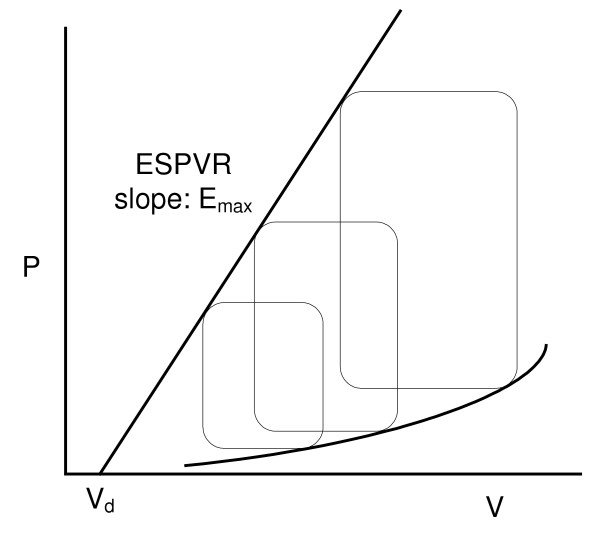
**Schematic drawing of P-V loops and end-systolic P-V relation (ESPVR)**. Schematic drawing of P-V loops and end-systolic P-V relationship (ESPVR) with a positive volume intercept V_d_. The slope of ESPVR line is deemed to be E_max_.

In this context, Suga [8] opted for the definition of ventricular elastance as



to represent the elastance of the contracting LV, where V_d _represents the unstressed LV volume corresponding to zero LV-pressure, obtained by drawing a tangent to P-V curves at end-ejection, as illustrated in Figure 1. This model gave rise to the development of the end-systolic pressure-volume relation (ESPVR) as a measure of contractility [9-14].

However, the determination of the maximal slope E_max _and of the volume-axis intercept (V_d_) of the tangent to the P-V curve at end-ejection (as a measure contractility of the cardiac muscle) is not only unreliable [11,15], but also requires generation of multiple P-V loops under variations loading conditions [11,15]. It is hence impractical to use clinically for a specific LV catheterization-ventriculography data. Above all, all of these variations in the concept of E obtained from LV pressure-volume data fail to explain the phenomena of LV suction and LV pressure drop during early filling as well as the generation of LV pressure increase during isovolumic contraction.

We have hence come up with a new concept of dual passive and active elastances operating throughout the cardiac cycle. The passive elastance (E_p_) represents the LV pressure response to LV volume change (to LV volume increase during LV filling phase and to LV volume decrease during LV ejection phase). However, simultaneously, we also have active elastance (E_a_) representing the contraction of the left ventricle due to its sarcomeric activation (and the development of force between the actin-myosin units) and relaxation (due to disengagement of the actin-myosin units).

LV E_a _develops after the start of isovolumic contraction, becomes maximum some time during late ejection and thereafter decreases and becomes zero during diastolic filling. On the other hand LV E_p _starts increasing after the initiation of LV filling as the LV volume increases. It reaches its maximum value at the end-of-filling phase, remains constant during isovolumic contraction, and thereafter decreases during ejection (as the LV volume decreases). While the generation of E_a _helps us to explain the development of the LV pressure increase during isovolumic contraction, the decrease of E_a _during diastole helps us to explain the decrease in LV pressure during early filling. The incorporation of both E_p _and E_a _helps us to explain the LV pressure changes during the filling and ejection phases.

## Methods

### Data acquisition

The subjects in this study were studied in a resting recumbent (baseline) state, after premedication with 100–500 mg of sodium pentobarbital by retrograde aortic catheterization. Left ventricular chamber pressure was measured by a pigtail catheter and Statham P23Eb pressure transducer; the pressure was recorded during ventriculography. Angiography was performed by injecting 30–36 ml of 75% sodium diatrizoate into the LV at 10 to 12 ml/s. It has been found by using biplane angiocardiograms that calculated orthogonal chamber diameters are nearly identical [16]. These findings are used to justify the use of single-plane cine techniques, which allow for beat-to-beat analysis of the chamber dimensions.

**Figure 2:**
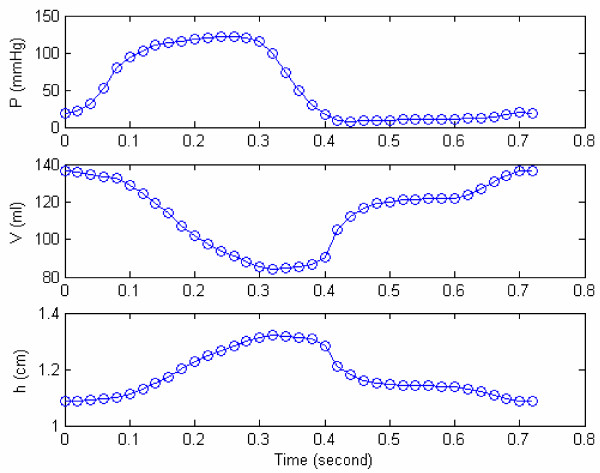
**A case study of measured LV pressure, volume and wall thickness during a cardiac cycle**. An example of a patient's measured LV pressure, volume and wall thickness during a cardiac cycle; t = 0-0.08s is the isovolumic contraction phase, t = 0.08s-0.32s is the ejection phase, t = 0.32s-0.40s is the isovolumic relaxation phase, and t = 0.40s-0.72s is the filling phase. Note that even after 0.4 s, the LV pressure still continues to decrease from 17 mmHg (at 0.4s, at start of filling) to 8 mmHg at 0.44s.

For our study, monoplane cineangiocardiograms were recorded in a RAO 30° projection from a 9 in image intensifier using 35 mm film at 50 frames/s using INTEGRIS Allura 9 system at the Nation Heart Centre (NHC), Singapore. Automated LV analysis was carried out to calculate LV volume and myocardial wall thickness. The LV data, derived from the cineangiographic films and depicted in Figure 2 consists of measured volume and myocardial thickness of the chamber as well as the corresponding pressure. All measurements are corrected for geometric distortion due to the respective recordings systems.

**Figure 3:**
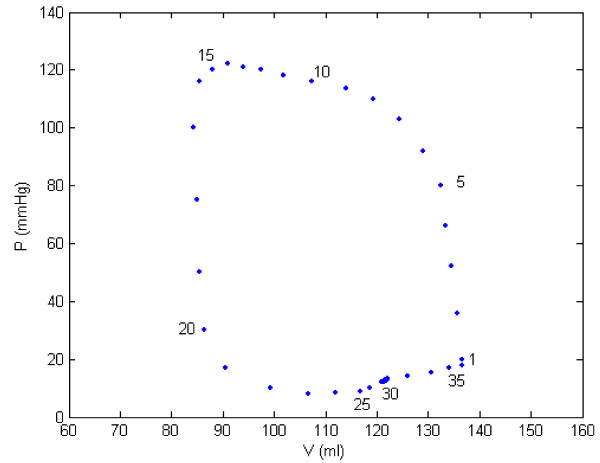
**Relationship between LV volume and pressure for the data of figure 2**. Relationship between LV volume and pressure for the data of Figure 1. Points (21–36) constitute the filling phase, (1–5) constitute the isovolumic contraction phase, (5–17) constitute the ejection phase, and (17–21) constitute the isovolumic relaxation phase. Note that after point 21, the LV pressure decreases; this characterizes LV suction effect.

In Figure 2, it is noted that during the early filling phase, LV pressure decreases even though LV volume increases. This phenomenon is defined as the 'LV suction effect', which will be explained later by using our concepts of active and passive elastances. This phenomenon is also depicted in Figure 3 and Table 1.

**Table 1:** Computed values of E_a _and E_p _during the cardiac cycle, for subject HEL

**Point**	**Phase**	**Time**	**Pressure**	**Volume**	**E_a_**	**E_p_**
1	Isovolumic contraction	0	18	136.7	0	0.968314
2		0.02	22	135.7	0.051287	0.930811
3		0.04	32	134.6	0.153167	0.891234
4		0.06	52	133.5	0.282331	0.853339
5		0.08	80	132.5	0.424912	0.820289

6	Ejection	0.1	94	129	0.570602	0.714374
7		0.12	103	124.5	0.711775	0.598039
8		0.14	110	119.3	0.843116	0.486996
9		0.16	113	114	0.961299	0.395008
10		0.18	116	107.3	1.06464	0.303159
11		0.2	118	101.8	1.15275	0.243961
12		0.22	120	97.5	1.22616	0.205852
13		0.24	121	94	1.28607	0.179272
14		0.26	122	91	1.334	0.159239
15		0.28	120	88	1.37114	0.141444
16		0.3	116	85.5	1.34002	0.128144
17		0.32	100	84.3	1.15107	0.122212

18	Isovolumic relaxation	0.34	74	85	0.846411	0.125638
19		0.36	50	85.5	0.523931	0.128144
20		0.38	30	86.4	0.269515	0.132782
21		0.4	17	90.6	0.113989	0.156743

22	Filling	0.42	10	105	0.0392726	0.276831
23		0.44	8	112	0.0109316	0.365003
24		0.46	8.4	117	0.00244008	0.444703
25		0.48	9	119	0.00043378	0.481259
26		0.5	9.6	120.2	6.10272e-005	0.50462
27		0.52	10.2	121	6.75442e-006	0.520821
28		0.54	10.5	121.4	5.84848e-007	0.529115
29		0.56	10.7	121.6	3.94098e-008	0.533312
30		0.58	10.8	121.8	2.0564e-009	0.537542
31		0.6	11	122	8.2698e-011	0.541805
32		0.62	11.8	124	2.55156e-012	0.586344
33		0.64	12.8	127	6.01397e-014	0.66011
34		0.66	14.5	130.7	1.07837e-015	0.763991
35		0.68	17	134	1.46518e-017	0.87036
36		0.7	20	136.6	1.50271e-019	0.964497
37		0.72	18	136.7	1.15909e-021	0.968314

### Definition of passive elastance and active elastance of the LV

At the start of diastolic-filling phase, the LV incremental pressure dP_LV _is the response to (i) LV E_a _continuing to decrease due to the sarcomere continuing to relax well into the filling phase, and (ii) to the rapid inflow of blood and the corresponding increase in LV volume, along with increase in LV E_p_. The associated governing differential equation, relating LV pressure and volume, can be put down (by referring to the Appendix for its derivation) as [17]:



where *t *represents the time variable (s) from the start of filling phase;

*V *represents the volume of LV (ml) during the filling phase;

*P*_*LV *_represents pressure of the LV, in mmHg (hereafter symbolized by *P*) (mmHg);

*M *represents the inertia term = [LV wall-density (*ρ*)/(LV surface-area/wall-thickness)] = *ρh */ 4*πR*^2^, for a spherical LV model (in mmHg/(ml/s^2^));

*E *represents LV elastance (mmHg/ml).

Likewise during ejection, the LV pressure variation (dP_LV_) is caused by both E_a _variation as well as E_p _decrease. The instantaneous time-varying ventricular elastance (E) is the sum of (i) volume-dependent passive elastance (E_p_) and (ii) active elastance (E_a_) due to the activation of the LV sarcomere. Hence,

*E *= *E*_*a *_+ *E*_*p *_    (6)

We will now provide the expressions for E_p _and E_a_, and then their formulations. The passive (unactivated) myocardium exhibits properties of an elastic material, developing an increasing stress as strain increases, as occurs during ventricular filling. The passive stress-strain relation of myocardial muscle strip is nonlinear, and therefore cannot be described by Hooke's law. As an approximation, it follows an exponential relationship [18-20]. Therefore, the relation between LV passive pressure and volume has also been assumed to be exponential. Since E_p _(= dP/dV) is volume-dependent, we can express it as:



where *E*_*p*0 _is the passive elastance coefficient, *z*_*p *_is the passive elastance exponent, and V is the LV volume; its derivation is provided in a subsequent section.

On the other hand, we will represent E_*a *_as an intrinsic property of the LV (derived later), as:



where (i) t is measured from the start of isovolumic contraction, (ii) the parameter *E*_*a*0 _is the active elastance coefficient, (iii) the time-coefficient (*τ*_*C*_) describes the rate of elastance rise during the contraction phase, while (*τ*_*R*_) describes the rate of elastance fall during the relaxation phase; (iv) the exponents "*Z*_*C*_" and "*Z*_*R*_" are introduced to smoothen the curvatures of the E_*a *_curve during isovolumic contraction and relaxation phases; (v) the parameter d is a time constant whose (to be determined) value is during the ejection phase, and (vi) u(t-d) is the unit step function, u(t-d) = 0 for t<d. The rationale for equation (8), as provided in the next section, is based on E_a _incorporating parameters reflecting the (i) generation of LV pressure during isovolumic contraction, (ii) decrease of LV pressure during isovolumic relaxation and early filling, and (iii) the LV pressure-volume relationship during filling and ejection phase.

### Our hypothesis

Based on equations (5–8), our hypothesis is that both E_a _and E_p _contribute to the relationship of LV pressure and volume. While E_p _incorporates LV pressure change due to LV volume change, E_a _incorporates the effect of LV myocardial activation in the generation of LV pressure during the isovolumic phases (when the LV volume remains constant). Since E_a _is deemed to be the basis of LV pressure generation, its variation (as given by equation 8) corresponds to the LV pressure variation.

### Determination of E_a _and E_p _expressions

#### a) Active elastance (during isovolumic contraction and relaxation)

During isovolumic contraction (because dV = 0,  and E_p _is constant), the governing equation (5) becomes *VdE *= *dP*_*LV*_, which can be detailed as:

*V*_*i*_(*E*_*i *_- *E*_*i*-1_) = *V*_*i*_[(*E*_*a,i *_+ *E*_*p,i*_) - (*E*_*a,i*-1 _+ *E*_*p,i*-1_)] = *V*_*i*_(*E*_*a,i *_+ *E*_*ped *_- *E*_*a,i*-1 _- *E*_*ped*_) = *dP*_*LV,i *_    (9)

where i is a time instant during the isovolumic contraction and relaxation, V_i _and P_LV,i _are the monitored LV volume and pressure at this instant, and E_ped _is the passive elastance at the end-diastolic phase.

Also, during isovolumic relaxation (because dV = 0,  and E_p _is constant), the governing equation (5) becomes *VdE *= *dP*_*LV*_, which can be represented as:

*V*_*i *_(*E*_*a,i *_+ *E*_*p,i *_- *E*_*a,i*-1 _- *E*_*p,i*-1_) = *V*_*i *_(*E*_*a,i *_+ *E*_*pes *_- *E*_*a,i*-1 _- *E*_*pes*_) = *dP*_*LV,i *_    (10)

where E_pes _is the passive elastance at the end-systolic phase

Now, applying equations (9 & 10) to the case shown in the Figure 2, we have (using the monitored LV pressure-volume data):

1. For isovolumic contraction

*E*_*a*,1 _= 0     (11-a)

*E*_*a*,2 _= (*P*_2 _- *P*_1_)/*V*_2 _+ *E*_*a*,1 _= 0.029477 mmHg/ml     (11-b)

*E*_*a*,3 _= (*P*_3 _- *P*_2_)/*V*_3 _+ *E*_*a*,2 _= 0.103771 mmHg/ml     (11-c)

*E*_*a*,4 _= (*P*_4 _- *P*_3_)/*V*_4 _+ *E*_*a*,3 _= 0.253584 mmHg/ml     (11-d)

*E*_*a*,5 _= (*P*_5 _- *P*_4_)/*V*_5 _+ *E*_*a*,4 _= 0.463599 mmHg/ml     (11-e)

2. For isovolumic relaxation

*E*_*a*,18 _= (*P*_18 _- *P*_17_)/*V*_18 _+ *E*_*a*,17 _= *E*_*a*,17 _- 0.058954 mmHg/ml     (11-f)

*E*_*a*,19 _= (*P*_19 _- *P*_18_)/*V*_19 _+ *E*_*a*,18 _= *E*_*a*,17 _- 0.177824 mmHg/ml     (11-g)

*E*_*a*,20 _= (*P*_20 _- *P*_19_)/*V*_20 _+ *E*_*a*,19 _= *E*_*a*,17 _- 0.312656 mmHg/ml     (11-h)

*E*_*a*,21 _= (*P*_21 _- *P*_20_)/*V*_21 _+ *E*_*a*,20 _= *E*_*a*,17 _- 0.463599 mmHg/ml     (11-i)

**Figure 4:**
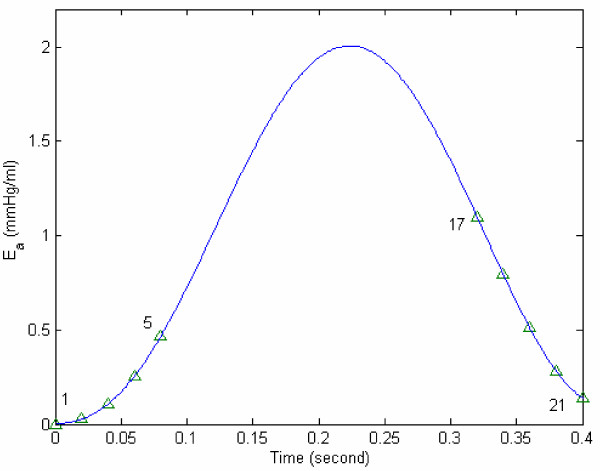
**E_a _vs. time for the data of figure 2. **Points (1–5) are the computed values of E_a _during isovolumic contraction phase, based on equations 11(a-e). In the isovolumic relaxation phase, the computed values are represented by the symbol Δ, for *E*_*a*,17 _= 1.1 *mmHg/ml*. The best fit for E_a _during isovolumic contraction and relaxation phase is given by the curve: *E*_*a *_= -12000*t*^6 ^+ 17000*t*^5 ^- 7700*t*^4 ^+ 1100*t*^3 ^+ 19*t*^2 ^+ 0.59*t *+ 0.00056



Now in the above expressions 11(f-i), E_a,17 _at end-ejection is unknown. For different representative values of E_a,17_, we can get different E_a _curves. We need to determine the optimal value of E_a,17_, such that E_a _can be described by a smooth curve to fit both isovolumic contraction and ejection phases. In Figure 4, we have determined E_a,17 _= 1.1 mmHg/ml, and the polynomial expression for E_a_(t) to fit its above calculated values during isovolumic contraction and relaxation. However, in order to (i) more suitably represent E_a _to correspond with its role during the cardiac phases (isovolumic, ejection and filling), and (ii) because of the sigmoidal shape of E_a _curve and its variation resembling the LV pressure variation during systole (as seen in Figure 4), we express E_a _(according to equation 8) as:

So that its constituent parameters have physiological significance as indicated following equation (8).

Hence, to compute E_a_(s) during isovolumic contraction, when u(t-d) = 0, we employ the expression



and determine its parameters E_a0_, *τ*_*C *_and Z_C _to fit the monitored pressure-volume data. Then, to compute E_a_(s) during isovolumic relaxation, we employ the total expression



and determine its remaining parameters d, *τ*_*R *_and Z_R _to fit the measured pressure-volume data.

**Figure 5:**
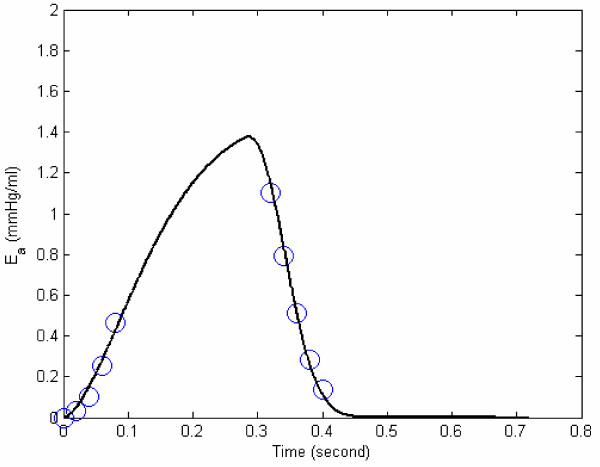
**The data of figure 2 is fitted with equation (8). **When the patient data of Figure 2 is fitted with equation (8), the resulting parameters values are obtained as: *E*_*a*0 _= 1.48 mmHg/ml, *τ*_*C *_= 0.1555 s, *Z*_*C *_= 1.631, d = 0.28 s, *τ*_*R *_= 0.07935 s, *Z*_*R *_= 2.267 s, *E*_*a*,17 _= 1.1 mmHg/ml, RMS = 0.026 mmHg/ml.

For the sample data of Figure 2, the variation of E_a _is depicted in Figure 5, along with the values of its parameters. We now propose that E_a _can be employed as an index of contractility.

#### b) Passive elastance determination during diastolic filling

During the diastolic filling phase, equation (5) becomes



**Figure 6:**
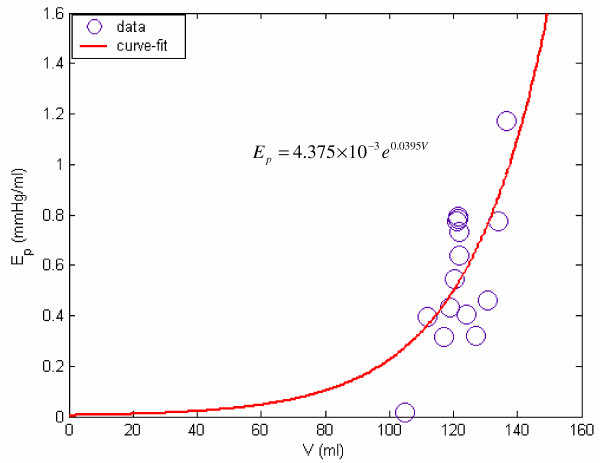
**Passive elastance E_p _vs LV volume for the data of figure 2**. Passive elastance E_p _vs LV volume V, for the sample case shown in Figure 2.

Now because E_p _is constant at a particular volume V_i_, equation (13) becomes





where i is a time-instant during diastolic filling, V_i _and P_LV,i _are the monitored LV volume and pressure at this time, and *M *= *ρh */ 4*πR*^2^. For the patient data (shown in Figure 2), we can get the mean value for M during diastolic filling, *M *= 8.03 × 10^-6 ^*mmHg/(ml/s^2^*). Therefore, from equation (15), we can calculate the values of E_p _at various instants during filling phase. We then plot E_p _vs V, in Figure 6. By fitting equation (7) to these calculated values of E_p_, we obtain the values of the parameters *E*_*p*0 _and *z*_*p*_, as:

*z*_*p *_= 0.0395 *ml*^-1^, *E*_*p*0 _= 4.375 × 10^-3 ^*mmHg */ *ml *    (16)

and the E_p _function (corresponding to its expression given by equation 7) as follows:

*E*_*p *_= 4.375 × 10^-3 ^*e*^0.0395*V *^    (17)

We now propose to adopt E_p _as a measure of LV resistance-to-filling.

Hence during ejection, both E_a _and E_p _are varying. During ejection and filling phases, E_p _can be calculated at any time using equation (17). Likewise, E_a _can be calculated during ejection and filling phases using equation (8), once its parameters have been determined by employing equation (12-b & 12-b) during isovolumic contraction and relaxation phases. Their values during the cardiac cycle are listed in Table 1.

## Clinical application results

The analyses, presented herewith, are now applied to clinically obtained data consisting of the subject's left ventricular (instant-to-instant) dimensions (obtained by cineangiocardiograph) and chamber pressure (obtained by cardiac catheterization). For each subject, passive and active elastances are determined from the left ventricular data. Table 2 provides the measured data and the model-derived parameters for three subjects (subject HEL, DDM, and ML). Subject HEL serves as a sample patient with myocardial infarct, subject DDM with double vessel disease (DVD) and hypertension, treated with PTCA; subject ML with idiopathic myocardial hypertrophy (IMH).

**Table 2:** Clinical history, measured hemodynamic data and calculated passive and active elastance parameters (E_p _and E_a_) for subjects (HEL, DDM and ML). Where LVP: left ventricle chamber pressure, AOP: aortic pressure, EDV: end-diastolic volume, ESV: end-systolic volume, EF: ejection fraction, MI: myocardial infarct, DVD: double vessel disease, HTN: hypertension, IMH: idiopathic myocardial hypertrophy, *E*_*a*,max _: maximum active elastance

**Subject**	**H.E.L**	**D.D.M**	**M.L**
Disease	MI, DVD	DVD, HTN	IMH
LVP (mmHg)	122/18	170/24	109/12
AOP (mmHg)	125/75	169/99	115/70
EDV/ESV (ml)	132.5/84.3	121.7/41.3	368/284
EF	0.36	0.66	0.23
*E*_*p*0 _(mmHg/ml)	4.375 × 10^-3^	6.74 × 10^-5^	1.442 × 10^-8^
*z*_*p *_(ml^-1^)	0.0395	0.07499	0.05024
*E*_*a*0 _(mmHg/ml)	1.48	4.4	0.595
*τ*_*C *_(s)	0.1555	0.207	0.1082
*Z*_*C*_	1.631	1.536	1.977
d (s)	0.28	0.26	0.18
*τ*_*R *_(s)	0.07935	0.1536	0.1377
*Z*_*R*_	2.267	2.943	1.873
*E*_*a*,max _(mmHg/ml)	1.37	3.58	0.57
*dP */ *dt*_max _(mmHg/s)	1200	1475	1125

**Figure 7:**
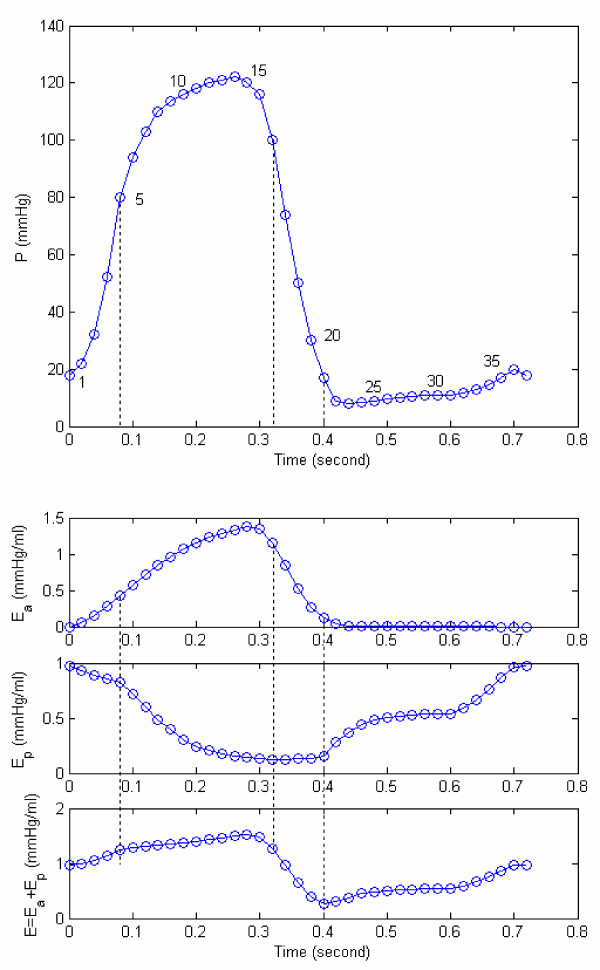
**Pressure, active elastance E_a_, and passive elastance E_p _and total E = E_a _+ E_p _for the data of figure 2. **Pressure, active elastance E_a_, passive elastance E_p_, and total *E *= (*E*_*a *_+ *E*_*p*_) for the sample subject shown in Figure 2. In this figure, 1–5 represents the isovolumic contraction phase, 5–17 represents the ejection phase, and 17–21 represents the isovolumic relaxation phase, 21–37 represents the diastolic filling phase.

**Figure 8:**
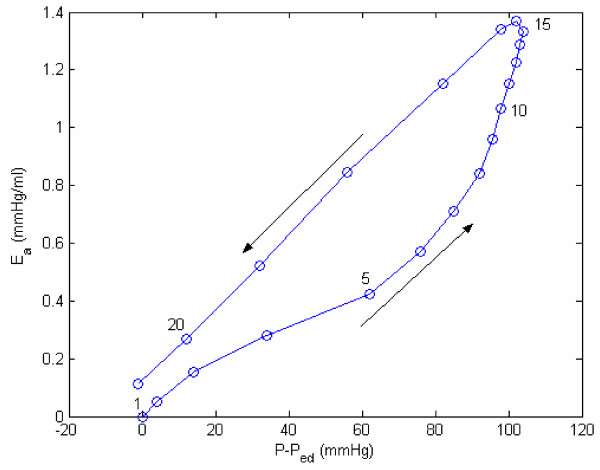
**Active elastance vs. incremental pressure. **Active elastance vs. incremental pressure (P-P_ed_) for the same subject as shown in Figure 2. The arrow direction indicates progression of time; 1–5: isovolumic contraction phase; 5–17: ejection phase. Note the rapid decrease in E_a _during the isovolumic relaxation that also extends into the filling phase, and causes suction of blood into the LV even before initiation of left-atrial contraction.

The variations of model-derived nonlinear passive and active elastances for the subject HEL are shown in Figure 7. For this particular subject (HEL), the maximum active elastance is 1.37 mmHg/ml. In Figure 8, we have plotted E_a _vs incremental pressure (P-P_ed_) for this patient HEL. Note that the elastance is much higher at late-ejection than early ejection. This is because of a continuing sarcomere stress development and shortening. The active elastance reaches its maximum value at late-ejection (point 15), and thereafter decreases. However as shown in Figure 7, even after the end of relaxation phase (point 21) the active elastance continue to decrease into the filling phase. This decrease can explain the suction effect during the rapid filling sub-phase, even after LV filling has commenced.

**Figure 9:**
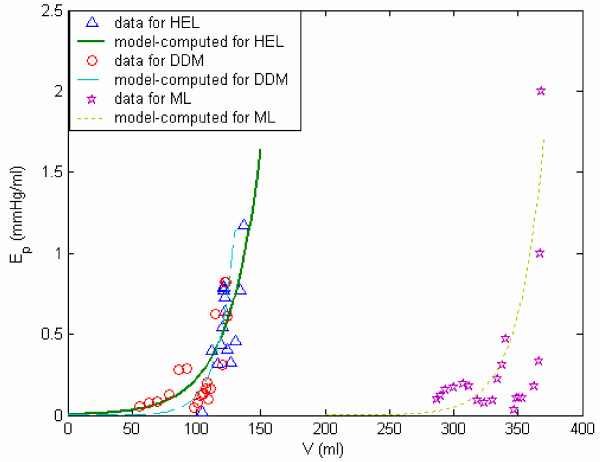
**LV volume and corresponding volume-dependent passive elastance. **Volume-dependent passive elastance (E_p_) for subjects HEL, DDM, and ML.

**Figure 10:**
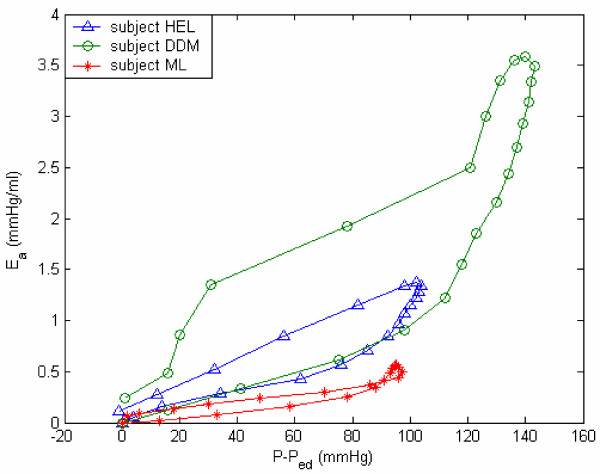
**Active elastance vs. incremental pressure. **Active elastance vs. incremental pressure for subjects HEL, DDM, and ML.

Figures 9 and 10 provide representations of the nonlinear passive and active elastance for subject HEL (with hypertension), DDM (myocardial infarct), and ML (idiopathic myocardial hypertrophy). The E_p _vs LV volume plots, in Figure 9, clearly reveal that E_p _increases exponentially with increase LV volume; the parameters (*E*_*P*0 _and *z*_*p*_) characterize this relationship. The passive elastance curve is steeper for a stiffer myocardium, with a corresponding bigger value of the exponential coefficient *Z*_*p *_(subject ML). The E_a _vs incremental pressure (P-P_ed_) plots, in Figure 10, reveal the development and decrease of E_a _during systole, which in turn governs the generation of LV pressure.

## Discussion

### Ea as a contractility index

**Figure 11:**
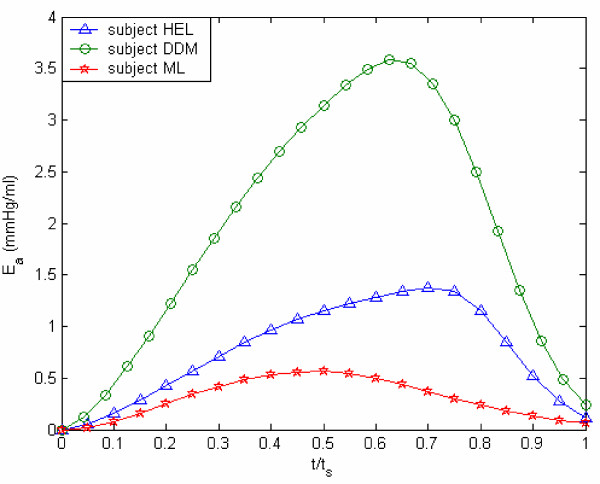
**Active elastance vs. normalized time. **Active elastance vs normalized time (t/t_s_) for subjects HEL, DDM, and ML. Herein, t_s _is the duration from start-of-isovolumic contraction phase to end-of-isovolumic relaxation.

**Figure 12:**
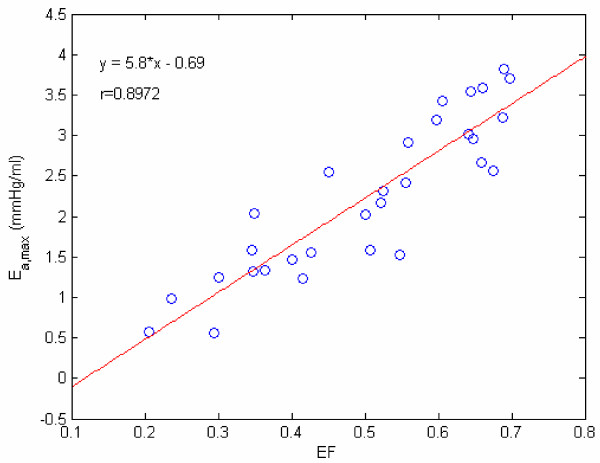
**E_a,max _vs EF. **Relating our contractility index E_a,max _to EF factor, with r being the correlation coefficient.

**Figure 13:**
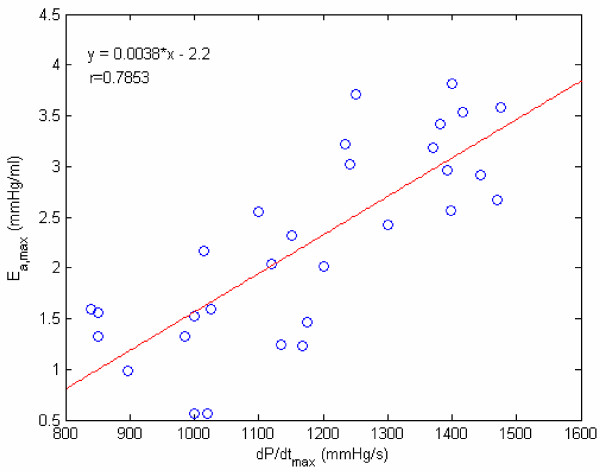
**E_a,max _vs dP/dt_max _.**Relating our contractility index E_a,max _to the traditional contractility index dP/dt_max_, with r being the correlation coefficient.

Yet another way to study E_a _variation is by means of the E_a _vs normalized time (t/t_s_) plot, shown in Figure 11. These two figures 10 and 11 make us realize that E_a,max _could be regarded as an index of LV contractility. Hence, we decided to plot E_a,max _vs the traditional contractility indices of EF and (dP/dt)_max_. These plots are displayed in Figures 12 and 13. It is noted that E_a,max _has a high degree of correlation with both EF and (dP/dt)_max_. It is interesting to compare our correlation-coefficient value (0.8972) with the value of 0.89 obtained by Mehmel et al [12], although this paper computes elastance as an extrinsic property = [P/(V-V_d_)]_es_.

### Demonstrating LV suction phenomenon

The active elastance curve can explain some critical LV physiological phenomena, namely LV pressure generation during isovolumic contraction and LV suction during early filling. The rapid decrease in elastance during isovolumic relaxation extends into the filling phase, and can explain the decrease in LV pressure (in Figure 7) even after LV filling has commenced. Let us show how this happens, by rewriting equation (15) as follows:

P_i _- P_i-1 _= (E_p,i _+ E_a,i_)(V_i _- V_i-1_) + V_i _(E_a,i _- E_a,i-1_)     (18)

by neglecting the  term, based on the calculation of its value being of the order of 10^-2 ^compared to (i) (*E*_*p,i *_+ *E*_*a,i*_)(*V*_*i *_- *V*_*i*-1_), which is of the order of 10^0 ^and (ii) *V*_*i*_(*E*_*a,i *_- *E*_*a,i*-1_) which is the order of 10^1^.

In equation (18), it is seen that P_i _can be less than P_i-1 _(or that P_i _- P_i-1 _< 0) only if (E_a,i _- E_a,i-1_) is negative, i.e., active elastance is decreasing. Now for subject H.E.L (Figure 1), the computed values of E_a _and E_p _at these 2 instants (based on equations 8 & 17) are:

*E*_*p*,22 _= 0.2768 *mmHg/ml*, *E*_*a*,22 _= 0.0393 *mmHg/ml*, *E*_*a*,21 _= 0.1140 *mmHg/ml*, *V*_22 _= 105 *ml*, *V*_21 _= 90.6 *ml *    (19)

Substituting these values into equation (18) gives (*P*_22 _- *P*_21_) = -3.22 *mmHg*, confirming decrease of pressure during early filling.

Hence, our novel concept of "decreasing E_a _during the early phase of filling" enables us to explain the phenomenon of decreasing LV pressure during the early stage of filling. In other words, it is suggested that the sarcomere actin-myosin activity continues into the filling phase. The decreasing E_a _during the filling phase seems to reflect decreasing sarcomeric activity during filling. Likewise, the increase in E_a _during isovolumic contraction is responsible for increase in LV pressure at constant volume, as demonstrated by means of equation (11).

### Demonstrating variation of LV pressure during ejection in terms of E_a _and E_p_

Similarly, both the active and passive elastances can explain LV pressure variation during ejection, using equation (18). Let us show how this happens by just taking two instants (t_10 _& t_9_), as follows:

P_10 _- P_9 _= (E_p,10 _+ E_a,10_)(V_10 _- V_9_) + V_10_(E_a,10 _- E_a,9_)     (20)

Substituting these computed values (listed in Table 1) into equation (20) gives *P*_10 _- *P*_9 _= 1.92 *mmHg*, which is approximately equal to the actual value of 2 mmHg.

## Concluding comments

Previous works have described elastance as a derived parameter from LV pressure-volume data, based on the definition of *P */ (*V *- *V*_*d*_) [9,10,12-14]. Also, E_max _(given by ESPVR) as illustrated on Figure 1 varies with different arterial loading states and is therefore load dependent [21], and not an intrinsic index independent of LV loading states.

Our definitions of E_p _and E_a _enable us to explain the phenomena of (i) LV suction during early filing, (ii) LV pressure rise during isovolumic contraction (iii) LV pressure variation during the ejection phase, and (iv) LV pressure drop during the relaxation phase. Both the concepts of E_a _and E_p _are made possible by our redefining elastance and compliance as

*dP *= *d(EV) *= *d(V/C) *= *VdE *+ *EdV *    (21)

Our concept of E_a _enables us to explain (i) the generation of pressure during isovolumic contraction and decrease of pressure during isovolumic relaxation, when the volume is constant, and (ii) the decrease of pressure during the rapid filling phase.

Our concept of decreasing E_a _along with increasing E_p _during filling, which enables us to explain the LV suction effect during early filling, is indirectly supported by the work of Shoucri [22]. He has indicated that the LV wall stress during the filling phase is made up of two components: (i) a passive increasing component due to LV pressure and (ii) an active decreasing component due to decreasing active fiber stress.

However, the determination of this active fiber stress in his paper is empirical and not based on a well-defined concept and expression for E_a_, as provided by us.

Finally, both E_a _and E_p _are invoked to explain the variations of pressure during the ejection and filling phases. From the viewpoint of intrinsic indices of LV assessment, E_p _can represent LV myocardial stiffness property and resistance to LV filling. On the other hand, E_a _has been shown to correspond to LV contractility, by means of Figures 12 & 13. Therein, we have shown a high degree of correlation between E_a,max _and (dP/dt)_max _as well as EF.

In future, we can also couple our LV model (of E_p _and E_a_) with an arterial lumped-parameter model (consisting of total peripheral resistance R, total arterial compliance C, aortic characteristic impedance Z_0 _and inertial L) in order to simulate and explain the mechanisms of chronic hypertensive states [23], in terms of alteration in E_*a *_itself as a measure of LV adaptation to chronic hypertension induced in the circulation system.

## Appendix: Derivation of Equation (5)

**Figure 14:**
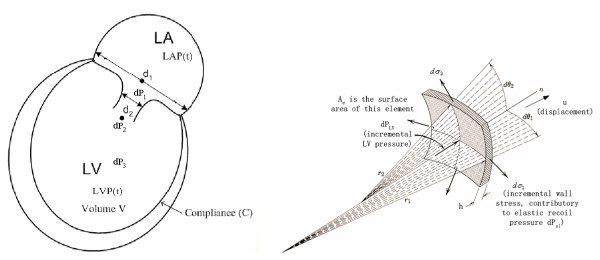
**Dynamic equilibrium of a myocardial element. **Dynamic equilibrium of a myocardial element. Element mass *m*_*e *_= *ρ*(2*r*_1_*dθ*_1_)(2*r*_2_*dθ*_2_)*h *= (*ρh*)(4*r*_1_*r*_2_*dθ*_1_*θ*_2_) = *ρhA*_*e *_= *m*_*s*_*A*_*e*_; *dσ*_*i *_= (*dP*_*el*_)(*r*_*i*_) / (2*h*), (i = 1, 2); *dP*_*el *_is the incremental elastic-recoil pressure. For dynamic equilibrium of the myocardial element, *m*_*e*_ + 2(*dσ*_1_)(2*r*_2_*dθ*_2_*h*) + 2(*dσ*_2_)(2*r*_1_*dθ*_1_*h*) - *A*_*e*_*dP*_*LV *_= 0, or *m*_*e*_ + *A*_*e*_*dP*_*el *_- *A*_*e*_*dP*_*LV *_= 0. For our LV spherical geometry model, r_1 _= r_2 _and *dσ*_1 _= *dσ*_2_.



Dynamic equilibrium of the LV myocardial element gives (based on figure 14):

where the myocardial element mass, *m*_*e *_= *m*_*s *_*A*_*e*_, *m*_*s *_(the myocardial surface-density or mass per unit surface area) = *ρh*, *ρ *is the myocardial density, and u is the radial displacement, *dP*_*el *_and *dP*_*LV *_are the incremental elastic-recoil and left ventricular pressures (as depicted in Figure A-1).

Now since,



By assuming *r*_1 _= *r*_2 _= *R*



we can write:



where *ρ*_*s *_is the surface density, *M *= *ρ*_*s*_*h*, and V is the LV volume. Now, referring to Figure A-1,



and



where *R*_*e *_is the resistance to LV filling (through the open mitral-valve), *ρ*_*f *_is the blood density and *v *is the blood velocity at site 2.



Let us now define incremental elastic recoil pressure (in response to incremental LV pressure *dP_*LV*_*) as:



Hence, from equations (A-1, A-4, A-7 & A-8), we have





Introducing the term elastance (= 1/C), we can put down



## Authors' contributions

Liang Zhong carried out the elastance studies, participated in data acquisition, performed statistical analysis and drafted the manuscript. Dhanjoo N. Ghista conceived of the study, and participated in its design and coordination and helped to draft the manuscript. Eddie Y-K Ng participated in coordination and helped to draft the manuscript. Soo T Lim participated in its design and coordination. All authors read and approved the final manuscript.
